# A call for bridging gender gap in HPV vaccination policies in Japan

**DOI:** 10.1002/hsr2.1421

**Published:** 2023-07-10

**Authors:** Yudai Kaneda, Mira Namba, Tshewang Gyeltshen

**Affiliations:** ^1^ School of Medicine Hokkaido University Sapporo Japan; ^2^ School of Medicine Keio University Tokyo Japan; ^3^ Graduate School of Public Health St. Luke's International University Tokyo Japan

In Japan, cervical cancer has the highest incidence rate among cancers affecting women aged 15–39, with human papillomavirus (HPV) infection reported as the primary cause.^1^ This situation can be traced back to 2013, when the Ministry of Health, Labor and Welfare (MHLW) ceased its active promotion of the HPV vaccine following reports of adverse events following immunization. Consequently, an 8‐year span was observed wherein the HPV vaccination rate lingered below 1%, which led to an estimated additional incidence of 24,600–27,300 cases and 5000–5700 mortalities from cervical cancer over the lifespan of cohorts born between 1994 and 2007.^2^ In response to this context, after its safety was confirmed, the Japanese government resumed active promotion of the HPV vaccine in November 2021 and, in April 2022, initiated HPV vaccination efforts targeting adolescent girls nationwide.^1^ Currently, girls aged 12–16 receive publicly‐funded vaccinations in a regimen of three doses, yet the prospect of reducing the number of doses to two or even one, in accordance with the World Health Organization's recommendation, remains unexplored. Additionally, from April 2022 to March 2025, a 3‐year HPV vaccination program is being implemented for women born between the fiscal years 1997 and 2006 who missed the opportunity to receive the vaccine during the suspension of public funding. However, although the main route of HPV transmission is considered to be skin‐to‐skin contact, and MHLW also previously announced the policy to include men in the HPV vaccine targets in November 2020,^1^ the specific efforts to address HPV infection in men are largely overlooked in Japan.

One compelling reason for men to receive the HPV vaccine is to prevent oropharyngeal cancer. Indeed, epidemiological statistics indicate that oropharyngeal cancers are the most common HPV‐associated cancer among men, with about 70% of the cases attributable to HPV infection.^3^ Over approximately 40 years, from 1975 to 2014, the incidence of oropharyngeal cancer in the United States increased by 57.3%,^4^ with similar trends observed among men in developed countries such as Australia, Canada, and Japan. Notably, the prevalence of oral HPV16/18/6/11 infections was higher among unvaccinated individuals; while it was 0% among vaccinated men, it was 2.1% among unvaccinated men, demonstrating a statistically significant difference.^5^ In response to these trends, efforts to promote HPV vaccination among men are being undertaken in various countries worldwide.

In the United States, the Centers for Disease Control and Prevention approved the vaccination for men in 2009, and since 2011, 11–12‐year‐old boys have been actively recommended for routine vaccination. Consequently, vaccination among adolescents progressed, and between 2013 and 2018, the vaccination rate for men aged 18–26 increased from 7.7% to 27.0%, reaching half of the rate for women (53.6%).^6^ Similarly, in the United Kingdom, starting in September 2019, 11–12‐year‐old boys have been included in the routine vaccination schedule, providing free vaccination up to the age of 25. Also, in Australia, HPV vaccination for 12–13‐year‐old girls were incorporated into the routine immunization schedule in 2007 and was expanded to include boys in 2013.

On the contrary, despite the Japanese government's declaration to include men in its HPV vaccination strategy in 2020, the actual implementation process is still insufficient; as it stands, it has yet to be included in the legally mandated vaccination protocol for men. The cost for the three‐dose HPV vaccination ranges approximately from 50,000 to 100,000 yen (350–700 USD) at medical institutions and is believed to be a significant barrier to vaccination for men. By making HPV vaccination accessible and inculcating it in routine immunization for men is a suggested solution. Indeed, some local governments such as Hirakawa City in Aomori Prefecture offer free vaccinations also for men, but nationwide efforts are still insufficient.

Additionally, it is considered that trusted individuals, such as primary care physicians, play a crucial role in recommending vaccination to increase HPV vaccination coverage among men. However, as the current situation resulting from the past suspension of active HPV vaccine promotion for women in Japan demonstrates, without routine vaccination, the number of medical institutions actively administering HPV vaccines to men may be limited. To protect both themselves and their partners from cancer, it is important for both men and women to receive the vaccine before reaching the age when the risk of HPV infection increases. Given the recent reconsideration of HPV vaccination for adolescent girls in Japan, we believe it is a public health imperative that similar considerations be extended to adolescent boys nationwide.

## AUTHOR CONTRIBUTIONS

**Yudai Kaneda**: Conceptualization; Data curation; Writing—original draft. **Mira Namba**: Writing—review & editing. **Tshewang Gyeltshen**: Writing—review & editing.

## CONFLICT OF INTEREST STATEMENT

The authors declare no conflicts of interest.

## TRANSPARENCY STATEMENT

Yudai Kaneda affirms that this manuscript is an honest, accurate, and transparent account of the research reported, that no important aspects of the research have been omitted, and that any differences from the planned research (and registration, if relevant) are explained by Yudai Kaneda.

## Data Availability

Data sharing is not applicable to this article as no new data were created or analyzed in this study.
